# Clinicians' confidence in diagnosing atypical anorexia nervosa: An experimental study of the role of patient and clinician characteristics

**DOI:** 10.1002/erv.3132

**Published:** 2024-08-20

**Authors:** Jessica Beard, Tracey D. Wade, Glenn Waller

**Affiliations:** ^1^ Department of Psychology University of Sheffield Sheffield UK; ^2^ College of Education, Psychology and Social Work Flinders University Adelaide South Australia Australia

**Keywords:** atypical anorexia nervosa, diagnostics, DSM‐5, UFED

## Abstract

**Objective:**

This experimental study investigated the weight loss parameters and resulting end weight that influence clinician confidence in diagnosing atypical anorexia nervosa (AAN).

**Methods:**

Clinicians (*N* = 67) read a series of vignettes where patient weight loss and end weight varied, then rated their confidence in an AAN diagnosis and alternatives. Using repeated measures ANOVAs, we examined patient (weight loss, end weight) and the possible relationship between clinician (e.g., age, profession) characteristics and confidence in diagnosing AAN.

**Results:**

Clinicians were most confident in an AAN diagnosis when patients had lost 10% or 15% of their body weight, leaving them at a high or normal weight. Clinicians considered 5% as significant weight loss for AAN, but only when the patient ended at a high or normal weight. However, they did not clearly differentiate AAN from unspecified feeding and eating disorder (UFED) when there was a 5% weight loss resulting in high or normal end weight. Clinician characteristics did not impact on confidence in diagnoses.

**Conclusion:**

The DSM's ‘significant weight loss’ criterion leaves room for clinicians to interpret it idiosyncratically. The lack of a clear pattern of UFED and AAN diagnosis has important clinical implications, as UFED tends to be dismissed as being less serious.

## Highlights

- Case vignettes were used to assess patient and clinician characteristics that impact clinician confidence in assigning an atypical anorexia nervosa (AAN) diagnosis.
- Clinicians were most confident in diagnosing AAN when patients had lost 10% or 15% of their body weight.
- The ‘significant weight loss’ criterion of DSM‐5 leaves substantial room for clinicians to interpret it idiosyncratically, which has implications for diagnosis, identified prevalence levels, and service demands.

## 1 INTRODUCTION

Atypical anorexia nervosa (AAN) was first introduced into the diagnostic and statistical manual of mental disorders (DSM‐5; American Psychiatric Association [APA], 2013) in response to problems identified in its previous iterations. Diagnostic criteria for AAN, however, were not well defined. To be diagnosed with AAN, all criteria for anorexia nervosa (AN) need to be met (restriction of intake, intense fear of weight gain, disturbance in the experience of weight and shape), but ‘despite significant weight loss, the individual's weight remains within or above the normal range’ (APA, 2013).

Not only does the DSM‐5 definition not provide guidance as to what constitutes ‘significant weight loss’, but neither does it stipulate when the weight loss should have occurred, or over what period. Similarly, there is no agreed upon definition in research (Forney et al., 2017). A recent systematic review found several areas of discrepancy in the operational definitions of AAN across studies (Harrop et al., 2021), including the amount of weight loss considered significant, and body mass index (BMI) cut‐offs. A further systematic review and meta‐analysis found that only three out of 24 studies investigating AAN reported criteria for ‘significant weight loss’ (Walsh et al., 2023).

Given that atypical eating disorder cases are common among adults (e.g., Fairburn et al., 2007; Helverskov et al., 2011) and between 34% and 43% of child and adolescent outpatient eating disorder treatment programme referrals are accounted for by AAN (Balzan et al., 2023; Forman et al., 2014), such inconsistencies matter. A poorly defined classification of AAN has implications on clinical practice. For example, a recent survey investigating the knowledge, attitudes, and current practices of adolescent care providers towards AAN relative to AN found that clinicians were: less likely to consider a diagnosis of AAN; less confident in identifying AAN and less likely to correctly identify AAN; less likely to refer patients with AAN to a specialist, and less likely to counsel patients with AAN on health risks (Kons et al., 2024). Furthermore, an unclear definition of significant weight loss has implications for diagnosis, identified prevalence levels, and service demands. For example, in an adolescent sample, Wade and O'Shea (2015) found an overall prevalence rate of 1.9% for AAN when using the DSM‐5 criterion of ‘significant weight loss’ (i.e., reduction of 1.3 kg/m^2^ in BMI), but found that an additional 4.7% of individuals might be diagnosed when removing this weight loss criterion. Most importantly, they found those without the weight loss demonstrated similar levels of impairment to the AAN group with the weight loss, as well as similar levels to those with full threshold eating disorder diagnoses (Wade & O'Shea, 2015). Thus, the boundary between the terms AAN and unspecified feeding and eating disorder (UFED) in DSM‐5 is also unclear.

The American Academy of Paediatrics (Kleinman & Greer, 2020), American Society for Parenteral and Enteral Nutrition (Bouma, 2017), Academy of Nutrition and Dietetics (White et al., 2012), and the Society for Adolescent Health and Medicine (Golden et al., 2022) recommend including percentage of weight loss in the assessment of malnutrition. Forney et al. (2017) assessed the point prevalence of AAN at > 5%, 10% and 15% weight loss respectively in a large non‐clinical sample. Fewer individuals were diagnosed with AAN as the weight loss criterion increased from 5% to 15%. Nonetheless, at all levels of weight loss, AAN was associated with higher levels of eating pathology and distress compared to non‐eating disorder controls. Therefore, even at just 5% weight loss, individuals with cognitive concerns (e.g., drive for thinness) have clinically meaningful eating pathology that differentiates them from controls.

Herb Neff et al. (2023) considered the same three weight suppression thresholds (5%, 10%, and 15%) among a group of Veterans with probable AAN and cognitive concerns. The difference in variance explained by the three weight suppression groups was small, but the 5% threshold explained most of the variance. This suggests that the exact percentage of weight suppression is less important than the presence of *any* weight suppression, and that even just 5% weight suppression could identify individuals with clinically significant eating pathology.

Whilst Forney et al. (2017) and Herb Neff et al. (2023) suggest 5% weight loss is significant, it remains unclear whether clinicians would recognise that significance at the time of diagnosis. It is possible that clinicians would be less likely to diagnose such individuals with AAN, instead placing them in DSM‐5's ‘residual’ category, UFED. This category aims to ‘catch’ all the presentations that do not fit into a particular eating disorder diagnosis (i.e., AN, bulimia nervosa [BN], binge eating disorder [BED], or other specified feeding and eating disorders). However, UFED tends to be dismissed as being less serious than other diagnostic categories, and is at risk of being overlooked when diagnoses are made (Wade & O'Shea, 2015). Errors of diagnosis could lead to inadequate clinical management of cases who have relatively high levels of eating pathology (Sawyer et al., 2016; Wade & O'Shea, 2015).

It is therefore important to understand what clinicians consider a ‘significant weight loss’, and how this definition influences how they diagnose AAN. A vignette study found that, despite vignettes describing identical behavioural and cognitive symptoms across each weight category, clinicians were more likely to identify a restrictive eating disorder when patients were considered to be below the average weight range for age and height compared to within or above the average weight range (Silbiger, 2024). It is therefore important to understand whether patient end weight after weight loss influences confidence in diagnosing AAN. Finally, it is also important to consider whether clinicians' own characteristics (e.g., age, profession, experience) play a role in their diagnostic practice, as such factors can influence clinical practice (Speers et al., 2022).

This experimental study had two aims. The primary aim was to investigate the weight loss parameters and resulting end weight/BMI that influence clinicians' confidence in using a diagnosis of AAN versus other diagnoses. The secondary, exploratory aim was to investigate whether those criteria interact with the clinicians' own characteristics (age, profession, experience). To address these aims, clinicians who work with eating disorders were asked to read a series of vignettes based on patients who vary in the amount of weight loss and in their end weight/BMI (high, normal/healthy, borderline low), and were asked to rate how confident they were that the patients would be diagnosed with AAN. Based on Forney et al.’s (2017) study, the impact of weight loss was considered at 0%, 5%, 10% or 15% of body weight. It was hypothesised that: (1) clinicians would be more confident in an AAN diagnosis when there has been a greater weight loss; and (2) this would be moderated by the patient's weight at the end of the weight loss. In addition, the potential role of clinician characteristics in determining confidence in AAN and other diagnoses will be explored (hypothesis 3).

## 2 METHODS

### 2.1 Design

The study used an experimental design, where all participants responded to vignettes representing clinical cases. The cases varied in their current BMI and the amount of weight loss to get to that BMI, but were otherwise comparable. In the primary analyses, the design had two within‐subject factors—current BMI and proportion of weight loss ‐ and the dependent variable was the clinician's confidence in different potential diagnoses (including AAN). Participant characteristics were used as between‐subject factors in the secondary, exploratory analyses. The study was approved by the University of Sheffield Ethics Committee (Ref. 051548) and was pre‐registered (https://osf.io/ht53c/).

### 2.2 Sample size analysis

An a priori sample size analysis was performed to determine the number of clinicians needed to assess the impact of patient weight loss and end weight on clinician confidence. Using G*Power 3.1.9.2, 62 clinicians were deemed necessary for a small effect size (*f* = 0.15), an alpha of 5% (two‐tailed) and power of 80%. The small effect size was set as there is no directly comparable literature to suggest an effect that is any larger. If a moderate effect size were assumed (*f* = 0.25), then the number of participants needed would be 24. This study recruited 67 clinicians who completed the study, and therefore was fully powered to detect small effect sizes in the ANOVAS.

The original pre‐registered plan was to consider a larger number of potential covariates. However, as the majority were non‐dimensional, the original plan for use of linear multiple regression proved inappropriate. Therefore, analyses were conducted with single covariates or additional independent variables, according to whether they were dimensional or categorical.

### 2.3 Participants

An opportunity sample of clinicians working with eating disorders in mostly public healthcare settings were recruited through professional contacts and through advertisements to international professional organisations and societies (Academy for Eating Disorders; Eating Disorders Research Society). They were given a link in the recruitment advertisement to an online information sheet and consent form. Those who chose to take part in the study continued to the online Qualtrics questionnaire, which they completed anonymously. Clinicians were not offered any incentives to take part in the study.

### 2.4 Measures and procedures

Twelve short case vignettes (Supporting Information S1) were used to determine the impact of varying patient weight loss and end weight on clinician confidence in diagnosis of AAN. The vignettes were brief to mimic the level of information a clinician might receive in an initial referral for assessment of an adult with an eating disorder. The vignettes varied by the percentage of weight lost (0%, 5%, 10% or 15%) and the patient's end weight after the loss (high: BMI 35.9–39.5; normal/healthy: BMI 20.5–24.3; borderline low: BMI 18.5–18.6). All other patient variables remained the same or similar (i.e., gender, age, duration of problem, eating behaviours and cognitions) to ensure that any differences in diagnostic confidence were due only to the influence of weight loss and end weight.

For the primary analyses, clinicians were asked to read all 12 vignettes (presented in random order), then rate how confident (0%–100%) they were that the patient in each vignette would be diagnosed with AN, AAN, BN, Subthreshold BN (BN of low frequency, and/or limited duration), BED, UFED, or no eating disorder. For this study, only percentage confidence in AN, AAN and UFED were used, because they were the most closely reflected in the vignettes, and the most likely to be overlapping in diagnostic practice (as outlined in the Introduction). Clinicians were asked to make their confidence ratings total 100% (e.g., 70% confidence in AAN, 30% confidence in AN).

For the secondary analyses, clinicians were also asked to complete demographic information (age, gender, ethnicity, profession, years spent in profession, years spent treating eating disorders). This information was collected to assess the impact of clinician characteristics on confidence in different diagnoses.

### 2.5 Data analysis

Data were analysed using repeated measures ANOVAs, using SPSS v.29. The outcome variable was the percentage confidence (0%–100%) in each diagnosis given by clinicians. The independent variables were patient weight loss (0%, 5%, 10%, and 15%), patient end weight (high, normal, and borderline low), and clinician characteristics. For the primary analyses, three two‐way repeated measures ANOVAs were used to examine the overall effect of patient weight loss (four levels) and end weight (three levels) on clinician confidence in diagnosis of AAN, AN and UFED. Effect sizes are reported as partial eta^2^, where an effect size of *η* ^2^ = 0.01 is categorised as small, *η* ^2^ = 0.06 is moderate, and *η* ^2^ = 0.14 is large.

For the secondary analyses (hypothesis 3), clinician age, profession, years treating eating disorders, and years spent in profession were treated as dimensional characteristics, and used as covariates. Clinician gender and ethnicity were not considered, due to most of the sample being female and of White ethnicity. Profession was split into categories of ‘therapists’ (*N* = 42) versus ‘other’ (*N* = 25). ‘Therapists’ included clinical psychologists (*N* = 33) and eating disorder therapists (*N* = 8), and counsellors (*N* = 1). Clinical psychologists have a doctoral level degree, whereas eating disorder therapists do not, but they still deliver therapy (e.g., post‐graduate CBT trained). ‘Other’ professions included psychiatrists (*N* = 10), medical doctors (*N* = 3), (mental health) nurses (*N* = 4), dietitians (*N* = 1) (clinical) social workers (*N* = 2), occupational therapists (*N* = 1), assistant psychologists (*N* = 2), doctoral students (*N* = 1), and clinical psychology graduate students (*N* = 1). As this was a categorical independent variable, it was used as in a three‐way ANOVA (confidence in diagnosis as a product of weight loss category × end weight category × profession).

## 3 RESULTS

### 3.1 Clinician demographics

Sixty‐seven clinicians took part in the study. The mean age of clinicians was 44.3 years (SD = 14.0). Fifty‐five clinicians were female (82.1%), 11 (16.4%) were male, and one clinician (1.5%) did not wish to share their gender. Sixty‐two clinicians (92.5%) were of White ethnicity, three participants (4.5%) were of Asian ethnicity, one clinician (1.5%) was of Hispanic/Latinx ethnicity, and one clinician (1.5%) did not wish to share their ethnicity. Most clinicians were clinical psychologists (47.8%). The majority of clinicians' highest education level was a PhD (29.9%), followed by a professional doctorate (e.g., Clinical psychology doctorate; 22.4%), a Masters degree (20.9%), a post‐graduate diploma (e.g., CBT course; 16.4%), and a Bachelors degree (10.4%). The mean number of years spent in their profession was 16.1 (SD = 13.9), and the mean number of years spent treating eating disorders was 13.4 (SD = 13.5). Most clinicians worked in a public healthcare setting (58.2%), with the remainder working in private healthcare (16.4%) or a mix of both (25.4%).

### 3.2 Primary analyses: Impact of patient weight loss and end weight on confidence in diagnosis

Table 1 shows the ANOVA results, with main effects for *level of weight loss* and *end weight*, and the interaction effects for the two factors. Supporting Information S2 shows the mean percentage confidence in each diagnosis by varying weight loss and end weight.

**TABLE 1:** Main and interaction effects for weight loss and end weight on confidence in each diagnosis.

	*F*	(df)	*p*	*η*2
AAN				
Weight loss^a^	36.480	(2.1, 141.5)	<0.001	0.36
End weight^a^	8.618	(1.5, 98.1)	0.001	0.12
Weight loss × end weight	4.478	(6, 396)	<0.001	0.06
AN				
Weight loss^a^	11.398	(2.1, 141.7)	<0.001	0.15
End weight^a^	62.151	(1.2, 76.8)	<0.001	0.49
Weight loss × end weight^a^	6.147	(3.8, 250.5)	<0.001	0.09
UFED				
Weight loss^a^	36.618	(1.9, 122.5)	<0.001	0.36
End weight^a^	11.368	(1.5, 97.6)	<0.001	0.15
Weight loss × end weight^a^	1.624	(5.0, 332.1)	0.153	0.02

Abbreviations: AAN, atypical anorexia nervosa; AN, anorexia nervosa; UFED, unspecified feeding and eating disorder.

^a^ Mauchly's test of Sphericity was significant, degrees of freedom were corrected using the Greenhouse‐Geisser method.

There were significant main effects of weight loss and end weight on the percentage confidence in each diagnosis, meaning confidence in diagnosis varied depending on both the level of patient weight loss and their end weight. There was a significant interaction effect between the amount of weight loss and end weight on percentage confidence in AAN and AN diagnosis, but not in UFED diagnosis. For AAN, confidence increased with the level of weight loss, particularly for those who ended with a weight that was normal or higher ‐ a medium effect size interaction (partial *η* ^2^ = 0.06). The lower confidence in the diagnosis of AAN among those who lost more weight (particularly 10%–15%) and ended at a lower weight is likely to be explained by the higher confidence in a diagnosis of AN in those who ended at a low weight, regardless of level of weight loss (but particularly for those whose weight fell by 5% or more), with a medium effect size (partial *η* ^2^ = 0.09). It is noteworthy that there was still a notable level of confidence in the AAN diagnosis (c.25%–30%) even where there was no loss of weight and regardless of the end weight.

### 3.3 Pairwise comparisons within weight variables

**FIGURE 1:**
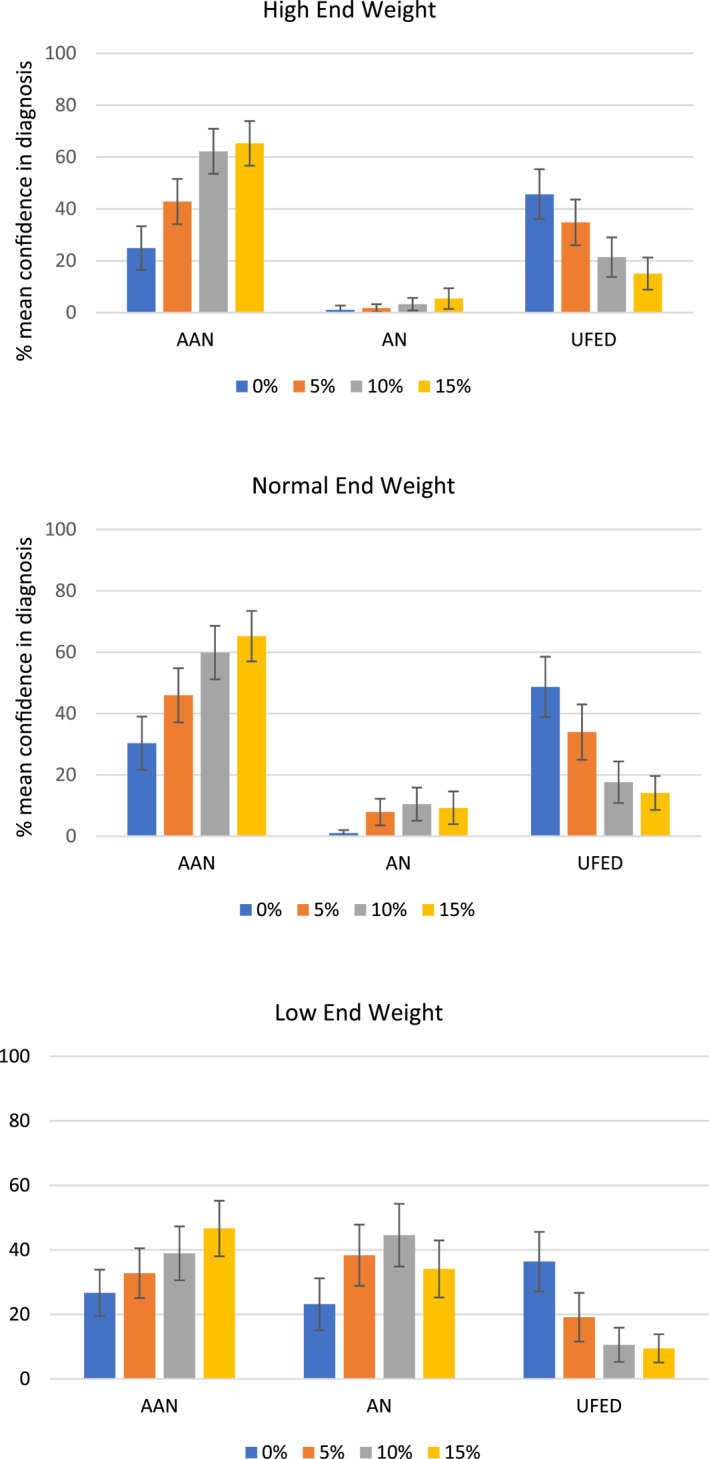
Clinician confidence (95% CI) in each diagnosis by end weight and weight loss.

To further explain the interaction effects between weight loss and end weight on clinician confidence, data were split by the three end weight groups. Figure 1 shows the mean confidence ratings and 95% confidence intervals for AAN, AN, and UFED at each end weight, according to level of weight loss. Tables 2, 3, 4 show pairwise comparisons for each group, based on the ANOVAS in Table 1 and the relevant mean scores. All pairwise comparisons were corrected (Bonferroni's) for multiple analyses.

**TABLE 2:** Percentage confidence in diagnoses at high weight by amount of weight loss.

Weight loss comparisons	Mean difference	95% CI	*p*
AAN			
0% versus 5%	−17.92	−28.86 to −6.98	<0.001
0% versus 10%	−37.30	−50.25 to −24.36	<0.001
0% versus 15%	−40.36	−54.41 to −26.32	<0.001
5% versus 10%	−19.38	−29.42 to −10.47	<0.001
5% versus 15%	−22.44	−34.42 to −9.35	<0.001
10% versus 15%	−3.06	−12.27 to 6.16	1.000
AN			
0% versus 5%	−0.64	−2.12 to 0.84	1.000
0% versus 10%	−2.16	−4.76 to 0.43	0.158
0% versus 15%	−4.33	−9.37 to 0.71	0.136
5% versus 10%	−1.52	−4.01 to 0.97	0.607
5% versus 15%	−3.69	−8.45 to 1.08	0.235
10% versus 15%	−2.16	−6.64 to 2.31	1.000
UFED			
0% versus 5%	10.86	−0.57 to 22.28	0.072
0% versus 10%	24.28	10.96 to 37.59	<0.001
0% versus 15%	30.62	16.81 to 44.44	<0.001
5% versus 10%	13.42	2.65 to 24.18	0.007
5% versus 15%	19.76	8.97 to 30.55	<0.001
10% versus 15%	6.34	−1.73 to 14.41	0.217

Abbreviations: AAN, atypical anorexia nervosa; AN, anorexia nervosa; UFED, unspecified feeding and eating disorder.

**TABLE 3:** Percentage confidence in diagnoses at normal weight by amount of weight loss.

Weight loss comparisons	Mean difference	95% CI	*p*
AAN			
0% versus 5%	−15.66	−28.51 to −2.80	0.009
0% versus 10%	−29.55	−45.21 to −13.90	<0.001
0% versus 15%	−34.93	−49.59 to −20.26	<0.001
5% versus 10%	−13.9	−26.27 to −1.52	0.019
5% versus 15%	−19.27	−31.76 to −6.78	<0.001
10% versus 15%	−5.37	−15.94 to 5.20	1.000
AN			
0% versus 5%	−6.85	−12.35 to −1.35	0.007
0% versus 10%	−9.40	−16.15 to −2.66	0.002
0% versus 15%	−8.21	−14.81 to −1.61	0.007
5% versus 10%	−2.55	−9.26 to 4.16	1.000
5% versus 15%	−1.36	−8.03 to 5.32	1.000
10% versus 15%	1.19	1.41 to −2.64	1.000
UFED			
0% versus 5%	14.70	2.82 to 26.58	0.008
0% versus 10%	31.05	17.91 to 44.18	<0.001
0% versus 15%	34.55	21.38 to 47.72	<0.001
5% versus 10%	16.34	4.33 to 28.36	0.003
5% versus 15%	19.85	8.07 to 31.63	<0.001
10% versus 15%	3.51	−3.44 to 10.46	1.000

Abbreviations: AAN, atypical anorexia nervosa; AN, anorexia nervosa; UFED, unspecified feeding and eating disorder.

**TABLE 4:** Percentage confidence in diagnoses at borderline low weight by amount of weight loss.

Weight loss comparisons	Mean difference	95% CI	*p*
AAN			
0% versus 5%	−6.14	−15.91 to 3.63	0.552
0% versus 10%	−12.26	−24.85 to 0.33	0.061
0% versus 15%	−19.98	−34.97 to −4.98	0.003
5% versus 10%	−6.12	−16.69 to 4.45	0.721
5% versus 15%	−13.84	−26.31 to −1.37	0.022
10% versus 15%	−7.72	−19.42 to 3.98	0.464
AN			
0% versus 5%	−15.17	−26.21 to −4.13	0.002
0% versus 10%	−21.41	−33.33 to −9.49	<0.001
0% versus 15%	−10.95	−24.29 to 2.40	0.174
5% versus 10%	−6.24	−14.31 to 1.83	0.235
5% versus 15%	4.22	−7.49 to 15.94	1.000
10% versus 15%	10.46	0.62 to 20.31	0.031
UFED			
0% versus 5%	17.22	5.63 to 28.81	<0.001
0% versus 10%	25.79	13.23 to 38.34	<0.001
0% versus 15%	26.90	14.01 to 39.77	<0.001
5% versus 10%	8.57	−0.56 to 17.70	0.028
5% versus 15%	9.67	0.67 to 18.67	0.015
10% versus 15%	1.10	−4.23 to 6.44	1.000

Abbreviations: AAN, atypical anorexia nervosa; AN, anorexia nervosa; UFED, unspecified feeding and eating disorder.

#### 3.3.1 Confidence in diagnosis for patients with a higher end weight

These analyses consider whether confidence in AAN, AN, or UFED diagnosis is related to level of percentage weight loss when the result is a high end weight. Table 2 shows the mean difference in confidence between weight loss comparisons for those patients who ended at a higher weight (see the first graph in Figure 1). Confidence in AAN was significantly higher when patients had lost 5% (*M* = 42.86, SE = 4.38), 10% (*M* = 62.24, SE = 4.35), or 15% (*M* = 65.30, SE = 4.31) of their body weight compared to having lost no weight (*M* = 24.93, SE = 4.23). Confidence in an AAN diagnosis was significantly higher when patients had lost 10% or 15% of their body weight compared to 5%. There was no significant difference in confidence in AAN diagnosis when patients had lost 10% versus 15% of their body weight. Confidence in AN did not differ significantly between any level of weight loss. Confidence in UFED was significantly higher when there was no weight loss (*M* = 45.70, SE = 4.82) compared to 10% (*M* = 21.42, SE = 3.83) or 15% (*M* = 15.07, SE = 3.09) weight loss, and significantly higher when there was 5% (*M* = 34.84, SE = 4.42) compared to 15% weight loss.

#### 3.3.2 Confidence in diagnosis for patients with a normal end weight

These analyses consider whether confidence in AAN, AN, or UFED diagnosis is related to level of percentage weight loss, when the result is a normal end weight. Table 3 shows the mean difference in confidence between weight loss comparisons for those patients who ended at a normal weight (second graph in Figure 1). Confidence in AAN was significantly higher when patients had lost 5% (*M* = 45.96, SE = 4.42), 10% (*M* = 59.85, SE = 4.37) or 15% (*M* = 65.22, SE = 4.12) of their body weight compared to 0% (*M* = 30.3, SE = 4.35). Confidence in AAN was significantly higher when patient had lost 10% or 15% of their body weight compared to 5%. Confidence in AAN did not differ significantly when the patient had lost 10% or 15%. Confidence in AN was significantly higher when patients had lost 5% (*M* = 7.90, SE = 2.17) 10% (*M* = 10.45, SE = 2.70) or 15% (*M* = 9.25, SE = 2.68) compared to no weight loss (*M* = 1.04, SE = 0.47). No other significant differences were found. Confidence in UFED was significantly higher when patients had lost no weight (*M* = 48.66, SE = 4.93) compared to 5% (*M* = 33.96, SE = 4.51), 10% (*M* = 17.61, SE = 3.40) or 15% (*M* = 14.10, SE = 2.78) of their weight, and when patients had lost 5% (*M* = 33.96, SE = 4.51) compared to 10% or 15%.

#### 3.3.3 Confidence in diagnosis for patients with a borderline lower end weight

These analyses consider whether confidence in AAN, AN, or UFED diagnosis is related to level of percentage weight loss, when the result is a borderline low end weight. Table 4 shows the mean difference in confidence between weight loss comparisons for those patients who ended at a lower weight (final graph in Figure 1). Confidence in a diagnosis of AAN was significantly higher when patients had lost 15% (*M* = 46.64, SE = 4.31) of their body weight compared to no weight loss (*M* = 26.67, SE = 3.61), and when patients had lost 15% compared to 5% (*M* = 32.81, SE = 3.87). No other differences were significant. Confidence in AN diagnosis was significantly higher when patients had lost 5% (*M* = 38.33, SE = 4.75) or 10% (*M* = 44.57, SE = 4.87) weight compared to no weight loss (*M* = 23.16, SE = 4.03). Confidence in AN was significantly higher when the patient had lost 10% of their body weight compared to 15% (*M* = 34.10, SE = 4.44). No other differences were significant. Confidence in UFED was significantly higher when there was no weight loss (*M* = 36.37, SE = 5.62) compared to 5% (*M* = 19.15, SE = 3.78), 10% (*M* = 10.58, SE = 2.65) or 15% (*M* = 9.48, SE = 2.2) weight loss. Confidence in UFED was significantly higher where there was 5% weight loss compared to 10% or 15% weight loss.

#### 3.3.4 Summary of impact of patient weight loss and end weight on confidence in diagnosis

Confidence in AAN was highest when patients had lost 10% or 15% of their body weight, resulting in high or normal weight after the weight loss. In contrast, confidence in AN was highest when there were similar levels of weight loss, but the result was borderline low weight. However, when patients lost any amount of weight resulting in borderline low weight, clinicians showed no difference in confidence in an AN versus an AAN diagnosis (no weight loss: *F*(1) = 0.343, *p* = 0.560; 5%: *F*(1) = 0.541, *p* = 0.465; 10%: *F*(1) = 0.436, *p* = 0.511; 15%: *F*(1) = 2.383, *p* = 0.127 respectively). Thus, when the patient was at a borderline low weight, clinicians did not clearly differentiate AN and AAN at any weight loss, including when there was no weight loss at all. Confidence in UFED was highest when patients had lost no weight, resulting in high or normal weight after the weight loss.

Confidence in UFED was significantly higher than confidence in AAN at a high (*F*(1) = 6.977, *p* = 0.010) and normal end weight (*F*(1) = 4.653, *p* = 0.035) when there was no weight loss, but was not significantly different at a borderline low end weight (*F*(1) = 1.876, *p* = 0.175). At 5% weight loss, the difference in confidence between UFED and AAN was not significant at a high end weight (*F*(1) = 1.059, *p* = 0.307) or a normal end weight (*F*(1) = 2.084, *p* = 0.154), but confidence in AAN was significantly higher than confidence in UFED where the end weight was borderline low (*F*(1) = 5.126, *p* = 0.027). Thus, clinicians did not clearly differentiate between AN and UFED when patients lost 5% of their body weight resulting in a high or normal end weight.

### 3.4 Secondary analyses: Impact of clinician characteristics on confidence in diagnosis

The earlier ANOVAs were repeated, taking account of the clinician characteristics that had previously been identified as potentially relevant. In the case of dimensional characteristics (age, duration qualified, duration working in the field of eating disorders), those characteristics were included as covariates. In the case of categorical features (e.g., profession), this was treated as an additional independent variable (meaning that the sample size was less able to identify small effect sizes).

#### 3.4.1 Categorical variable

Clinician profession (psychological therapists vs. others) had no significant impact on confidence in diagnosis of AAN (*F*(6) = 1.472, *p* = 0.186), AN (*F*(3.80) = 0.740, *p* = 0.559) or UFED (*F*(5.03) = 0.942, *p* = 0.454).

#### 3.4.2 Dimensional variables

Due to violations of the sphericity assumption, the Greenhouse‐Geiser statistic was used. Initially, judgements regarding **AN** were examined. For *clinician age*, there was a significant interaction of level of weight loss × end weight, even when age was controlled for (*F*[6390] = 3.89; *p* < 0.03), with a small‐medium effect size (partial *η* ^2^ = 0.041). The same pattern was found for *years treating eating disorders*, where the interaction of weight loss and end weight was significant when time treating eating disorders was accounted for (*F*[6390] = 3.15; *p* < 0.02), with a small‐medium effect size (partial *η* ^2^ = 0.046). Finally, the same pattern was found for the third temporal variable, *years in profession*, where the interaction of weight loss and end weight was significant when years qualified was accounted for (*F*[6390] = 2.46; *p* < 0.05), with a small‐medium effect size (partial *η* ^2^ = 0.036).

There was a broadly comparable pattern when considering **AAN** as the potential diagnosis. The outcomes were the same for *years treating eating disorders* (*F*[6390] = 3.17; *p* < 0.01), with a small‐medium effect size (partial *η* ^2^ = 0.046) and for *years in profession* (*F*[6390] = 2.60; *p* < 0.03), with a small‐medium effect size (partial *η* ^2^ = 0.038). However, the interaction was no longer significant when age was the covariate (*F*[6390] = 1.73; *NS;* partial *η* ^2^ = 0.026).

Finally, there was a different pattern when considering likelihood of **UFED** as a potential diagnosis. In no case was there an interaction of weight loss × end weight (*F* < 2.00, *NS* in all cases), and there was no covariate effect of any of the three temporal variables.

To summarise, weight loss and end weight interacted in how confident clinicians were about diagnosing patients with AN or with AAN. However, this was not the case for UFED. However, in no case did any of the three temporal factors influence that interaction.

## 4 DISCUSSION

This study aimed to examine patient and clinician characteristics that influence clinician confidence in diagnosing AAN. Clinicians read a series of vignettes where patient weight loss and end weight varied, then rated their confidence in an AAN diagnosis and alternatives. Consistent with the first two hypotheses, clinicians were most confident in an AAN diagnosis when patients had lost 10% or 15% of their body weight, leaving them at a high or normal weight after the weight loss. There was evidence to suggest that clinicians consider 5% weight loss as ‘significant’ (in keeping with Forney et al. (2017) and Herb Neff et al. (2023)), but only when the patient ended at a high or normal weight. However, clinicians did not clearly differentiate AAN from UFED when there was only a 5% weight loss at a high or normal end weight. It is also noteworthy that clinician confidence in AN and AAN diagnoses did not differ significantly at any amount of weight loss (0%, 5%, 10%, 15%) in cases where the patient ended at a borderline low weight.

Considering the third, exploratory hypothesis, the investigated clinician characteristics did not have a significant influence on confidence in AAN or UFED diagnosis. Such diagnostic confidence was not related to temporal factors or to therapist status, suggesting that it is a robust characteristic.

Broadly, this experimental study has confirmed that there is inconsistency in how clinicians interpret the weight loss criteria that DSM‐5 suggests for AAN (Wade & O'Shea, 2015). Rather than seeing categorical changes in confidence at specific weight criteria, confidence shows more of a linear relationship with weight loss, moderated by the patient's resulting weight level. However, there was still a notable level of confidence in AAN (c.25%–30%) even in the absence of weight loss, regardless of end weight. This supports Johnson‐Manguia et al. (2024), who found 64% of care providers in their study diagnosed AAN in the absence of any weight loss, provided other AN criteria were met. This finding suggests that the lack of a clear definition of AAN might result in substantial deviations from the DSM‐5 criteria.

The study highlights some key findings that warrant consideration for future research and clinical practice. First, clinicians may not be considering 5% as significant weight loss as a broad rule, despite evidence that such weight loss is clearly clinically significant (Forney et al., 2017; Herb Neff et al., 2023). Indeed, clinicians did not differ significantly in their confidence in an AAN or UFED diagnosis when the patient had lost 5% of their body weight, resulting in a high or normal end weight. This lack of clear differentiation between AAN and UFED in DSM‐5 and in clinical judgement risks inappropriately placing normal‐to‐high weight individuals with a 5% weight loss into the often‐overlooked UFED category, when such individuals can demonstrate the same levels of global eating disorder severity and impairment as other full and subthreshold eating disorders (Wade & O'Shea, 2015). Interestingly though, clinicians were significantly more confident in an AAN diagnosis compared to a UFED diagnosis when there was a 5% weight loss resulting in borderline low weight, suggesting clinicians might consider the patient's weight as more clinically relevant in differentiating AAN from UFED when there has been only a small percentage of weight loss.

Another notable finding worth further consideration is that clinicians were significantly more confident in an AN diagnosis at a borderline low weight when the patient had lost 10% compared to 15% of their body weight. It would be interesting to investigate confidence in each diagnosis with a 20% weight loss.

A final clinical consideration is that clinicians did not differ significantly in their confidence in AN and AAN diagnoses when patients had lost any amount of their body weight, where that loss resulted in a borderline low end weight. Research has shown that individuals with AN and AAN tend to experience similar, if not higher, levels of psychopathology and physiological complications (Fitterman‐Harris et al., 2024; Johnson‐Manguia et al., 2023; Walsh et al., 2023). It is therefore possible that clinicians in this study do not find the distinction between AN and AAN useful, and possibly would prefer to err on the side of caution with an AN diagnosis. Indeed, qualitative research suggests that clinicians find the AAN diagnosis stigmatising and that it is often not taken seriously relative to other eating disorders (Johnson‐Munguia et al., 2024). Further qualitative research into clinicians' perceived utility of the separate AN and AAN diagnoses would be beneficial. More research is needed into individuals at a borderline low weight to determine whether separating AN from AAN is clinically meaningful, and what this would mean in terms of accessing treatment. Critically, that distinction between the two needs to be codified in diagnostic practice, rather than expressed by the relatively vague ‘significant weight loss’ of DSM‐5. Clinicians need guidance as to what level of weight loss is likely to be meaningful, possibly using the 5% identified as clinically significant by Forney et al. (2017) and Herb Neff et al. (2023), or guidance around BMI in the presence or absence of starvation symptoms.

This study has some limitations that should be addressed in future research. First, it might be underpowered to detect small effect sizes in the analyses relating to the third, exploratory hypothesis. Thus, although no clinician characteristics were found to impact the confidence in diagnoses, this could be due to the smaller sample size. Similarly, a broader clinician group would help to determine whether characteristics (e.g., gender, ethnicity) play a role in clinician confidence in different diagnoses. Second, the vignettes varied only in the percentage weight loss and end weight. Future research should consider other factors that might bear on confidence in diagnosis (e.g., the period over which the weight loss occurred; how long ago the weight loss occurred; whether the patient is continuing to lose weight at presentation; impact on patient cognitions/psychopathology; gender and age of patient).

### 4.1 Clinical and service implications

In clinical practice, application of DSM‐5 criteria for AAN are intended to be linked to amount of weight loss and end weight status. However, these criteria clearly leave substantial room for clinicians to interpret them idiosyncratically. This ambiguity also appears to lead some clinicians to ignore the weight loss criterion altogether (Johnson‐Manguia et al., 2024). That is not to say that cases where there is little or no weight loss should be ignored, as they can have substantial clinical impairment (Wade & O'Shea, 2015). However, the tendency to treat UFED as less severe means that a misdiagnosis can have important clinical implications where there is substantial eating disorder pathology and associated impairment, regardless of weight loss. Clearly, errors in diagnosis of AAN have implications on effective treatment allocation, and indeed on future treatment developments for AAN. Furthermore, inconsistent application of the DSM‐5 criteria is likely to result in inaccurate data on the prevalence of AAN. Eating disorder services cannot know what demands will be made of them without knowing prevalence rates, and planning for that demand is dependent on good diagnostic practice.

## 5 CONCLUSION

There is clearly confusion based on existing diagnostic definitions for AAN. Three diagnostic adjustments can be suggested for future definitions. First, define what is meant by ‘significant weight loss’. Second, DSM‐5 criteria should be amended to reflect the clinical relevance of even relatively small amounts of weight loss. That level could be as low as 5% (Forney et al., 2017; Herb Neff et al., 2023). Guidance around the resulting weight after the loss should also be provided in order to allow AAN and AN to be differentiated. Third, and more radically, it could be argued that clinical practice should be defined in terms of clinical need, in keeping with Wade & O’Shea's (2015) summary. That might mean removing terms like AAN and UFED, and developing a more pathology‐defined grouping, based around eating‐disordered behaviours (e.g., food avoidance), cognitions (e.g., distress around body image and restrictive attitudes), and clinical levels of impairment to quality of life. Defining such a group positively (e.g., ‘eating distress’) rather than in the more conventional DSM manner (‘unspecified’) is likely to reduce the chances that clinicians and researchers will see these cases as being less important and as being less in need of effective interventions.

## AUTHOR CONTRIBUTIONS

All three authors conceptualized the paper and contributed to writing the final manuscript and approved the submitted version. Jessica Beard and Glenn Waller oversaw the data collection and conducted the data analyses.

## CONFLICT OF INTEREST STATEMENT

The authors declare no potential conflicts of interest.

## Supporting information

Supporting Information S1

Supporting information S2

## Data Availability

The data used are available on reasonable request to the corresponding author.
