# Supplementary material for: Clinicians' confidence in diagnosing atypical anorexia nervosa: An experimental study of the role of patient and clinician characteristics
Source: Eur Eat Disord Rev. 2024 Aug 20;33(1):106–17. doi: 10.1002/erv.3132 (PMC11617791; doi:10.1002/erv.3132)
Supplement: Supplementary file 1 — Supporting Information S1 [file ERV-33-106-s001.docx]

**Supplement A - Case Vignettes**

**No weight loss**

***High end weight***

Tara is a 20 year old female. When Tara started university at 19 she became worried about her weight and shape, reporting feeling fat compared to her housemates. As a result, she began restricting her daily food intake to one meal and one snack. She reports feeling terrified of gaining weight, as she already worries about how big people think she looks. Tara is obsessed with her weight and body shape, and thinks about this constantly. Tara has remained at a BMI of 39.5.

***Normal end weight***

Aisha is a 21 year old female. When Aisha left school at 18 she became worried about her weight and shape, reporting feeling fat compared to her university flatmates. She started eating only one main meal and snack each day. Aisha fears she will gain weight if she eats any more than this. She has become obsessed with her weight and looks, and thinks others talk about how big she is. Aisha has remained at a BMI of 24.3.

***Borderline low end weight***

Beatrice is a 22 year old female. When she started university at 20 she became distressed about her weight and appearance after her university friends made a joke about her shape. As a result, she reduced her daily food intake to one small meal and snack. Beatrice became obsessed with her weight and appearance and tried desperately to lose weight. She worries what other people think about her looks and size. Beatrice has remained at a BMI of 18.5.

**5% body weight loss**

***High end weight***

Hannah is a 21 year old female. At the age of 19, Hannah’s family made a comment about her weight. Ever since, she has become obsessed with trying to lose weight and terrified of gaining it. She hates the way she looks, and thinks that everyone else must think that she looks extremely overweight. To try to lose weight, Hannah began only eating one small meal and snack per day, avoiding carbs in both of these. Her current BMI is 35.9 down from 37.9 (a weight loss of 5.7kg/12.6lbs).

***Normal end weight***

Ese is a 21 year old female. At 18, Ese’s boyfriend made a comment about her appearance. Ever since, all she can think about is her weight and the fear of gaining any more. She became obsessed with trying to lose weight, eating only one meal and two snacks a day, avoiding fats in all of these. She hates the way she looks, and thinks that everyone else must also think looks overweight. Ese’s current BMI is 20.5 down from 21.9 (a weight loss of 3.2kg/7.1lbs).

***Borderline low weight***

Isabella is a 22 year old female. At 20, she became obsessed with controlling her weight after her friend told her she looks better with some more weight on her. Ever since, she started calorie counting and became frightened of gaining weight. She hates her appearance and worries about how big everyone must thing she is. To try to lose weight, Isabella started eating only one small meal and snack per day, avoiding carbs in both of these. Her current BMI is 18.5 down from 19.7 (a weight loss of 2.5kg/5.5lbs).

**10% body weight loss**

***High end weight***

Sofía is a 23 year old female. At the age of 21, Sofía became obsessed with her weight and appearance after being teased by her friends. She became scared of putting any weight on, focusing on trying to lose as much as possible by only eating a small meal and snack per day, avoiding fats in both of these. She hates her appearance and worries what everyone else thinks about her weight. Her current BMI is 40.9 down from 46.1 (a weight loss of 13.3kg/29.3lbs).

***Normal end weight***

Julie is a 24 year old female. At 22, Julie became worried about her weight and how she looks after being called fat by her ex-boyfriend. She became scared of putting on weight, and started trying to lose as much as possible. Julie began only eating two small meals per day, avoiding carbohydrates. She hates how she looks, and worries about what other people think of her in terms of her weight. Her current BMI is 21.1 down from 24.1 (a weight loss of 7kg/15.4lbs).

***Borderline low weight***

Priyanka is a 23 year old female. At the age of 20, she was teased by her friends for her appearance, and so became obsessed with her looks, weight and shape. She became terrified of gaining any weight, focusing on trying to lose as much as possible by restricting her intake to one small meal and two snacks per day. Priyanka hates the ways she looks and worries what everyone else must think of her. Her current BMI is 18.5 down from 20.5 (a weight loss of 5.7kg/12.6).

**15% body weight loss**

***High end weight***

Maya is a 22 year old female. At the age of 20, she became concerned about her weight after comparing herself to her flatmates. Since this, she has been desperate to lose weight, and started calorie counting every meal, which she restricted to one small snack and meal per day. Maya continues to compare herself to others and hates her appearance. She is concerned about what others think about her body. Her current BMI is 38.7 down from 46.1 (a weight loss of 19.7kg/43.4lbs).

***Normal end weight***

Beth is a 23 year old female. At 20, she started comparing herself to other women, and as a result became concerned about her weight and shape. Since this, she has tried desperately to lose weight. Beth began eating one small snack and meal per day, and calorie counts everything. Beth hates her weight and appearance and worries about what others think of her body. Her current BMI is 22.1 down from 25.4 (a weight loss of 10.8kg/23.8lbs).

***Borderline low weight***

Manuela is a 22 year old female. At the age of 19, she became concerned about her weight after being teased by her boyfriend for eating lots of snacks. Since this, she has restricted her food intake to try to control her weight. She now only eats one snack and two small meals per day, avoiding carbs in all of these. Manuela worries what other people think of her in terms of her weight and shape, and hates her appearance. Her current BMI is 18.6 down from 22.2 (a weight loss of 9.5kg/20.9lbs).
