# Supplementary figures and images for: Clinicians' confidence in diagnosing atypical anorexia nervosa: An experimental study of the role of patient and clinician characteristics
Source: Eur Eat Disord Rev. 2024 Aug 20;33(1):106–17. doi: 10.1002/erv.3132 (PMC11617791; doi:10.1002/erv.3132)

**Supplement B – Mean percentage confidence (95% CI) in each diagnosis by weight loss and end weight**

| 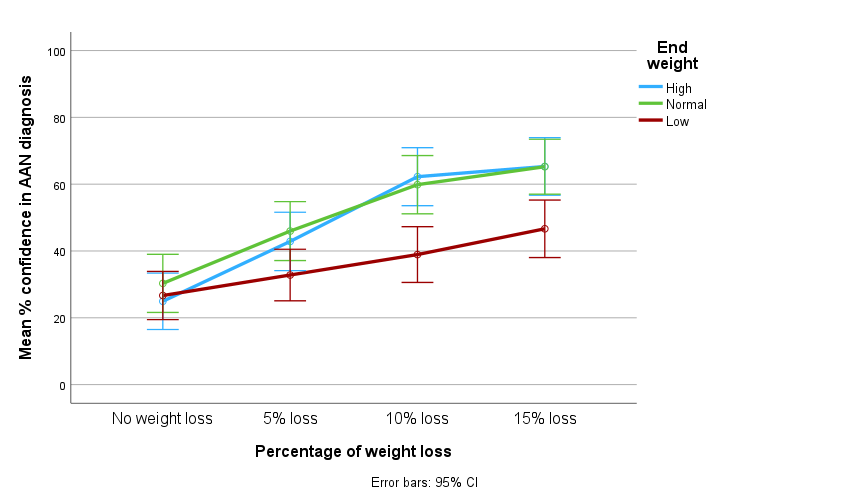 |
| --- |
| 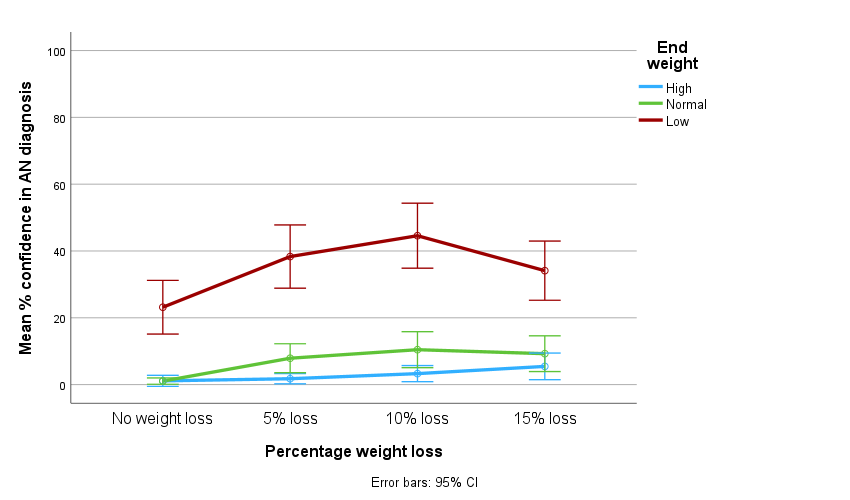 |
| 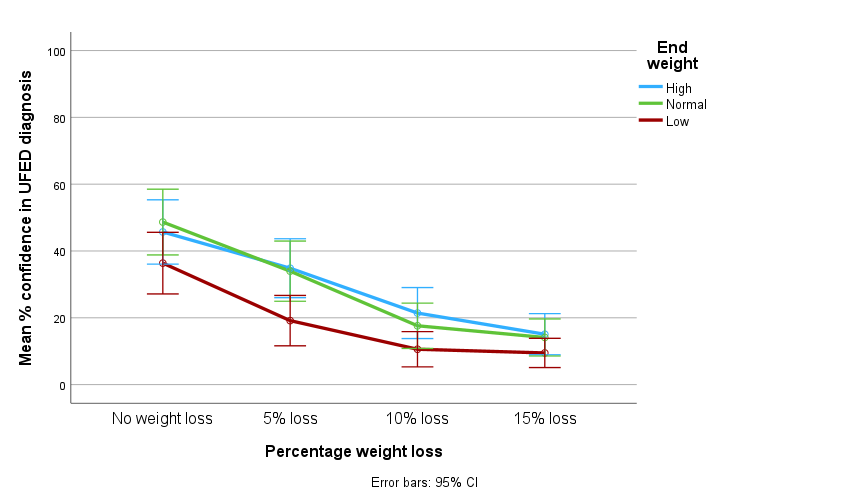 |

Supplement: Supplementary file 2 — Supporting information S2 [file ERV-33-106-s002.docx]
